# Exposure Scenarios for Estimating Contaminant Levels in Healthy Sustainable Dietary Models: Omnivorous vs. Vegetarian

**DOI:** 10.3390/foods13223659

**Published:** 2024-11-17

**Authors:** Helena Ramos, Ana Reis-Mendes, Marta Silva, Mafalda Ribeiro, Ana Margarida Araújo, Cristiane Borges, Olga Viegas, Armindo Melo, Zita Martins, Miguel A. Faria, Isabel M. P. L. V. O. Ferreira

**Affiliations:** 1 LAQV/REQUIMTE, Faculty of Pharmacy, University of Porto, 4050-313 Porto, Portugal; 2 Faculty of Food Science and Nutrition, University of Porto, 4099-002 Porto, Portugal; 3 Environmental Health Department, National Health Institute Dr. Ricardo Jorge, 4000-055 Porto, Portugal; 4 Associated Laboratory for Green Chemistry (LAQV), Network of Chemistry and Technology (REQUIMTE), 4050-313 Porto, Portugal

**Keywords:** FAIR database, food contaminants, dietary models, omnivorous diet, vegetarian diet, hazard assessment

## Abstract

Consumers are regularly exposed to well-known food contaminants (FCs), which are typically assessed for risk on an individual basis. However, there is limited knowledge about the overall levels and combinations of these compounds depending on dietary choices. The goal of this study was to estimate the real-life mixtures of FCs in different dietary models by integrating extensive data from the scientific literature concerning the reliable quantification of FCs in foods. A FAIR database detailing the occurrence of 73 FCs in 16 foods commonly consumed was built. The data were integrated into an omnivorous and a vegetarian dietary model. A weighted estimate of the 25th, 50th, and 75th percentiles of FCs in both dietary models revealed that the omnivorous model presented slightly higher levels of FCs than the vegetarian. At the 25th percentile, the FC levels in both dietary models fall within the European Food Safety Authority (EFSA) reference exposure levels for chemical hazards, except for arsenic, lead, cadmium, fumonisin B1, and OTA. At the 75th percentile, the FC levels exceed the EFSA reference levels for those FCs and additional mycotoxins. Using in vitro models, the 25th percentile can mimic real-life FC exposure, while the 75th percentile simulates a possible worst-case scenario.

## 1. Introduction

Consumers are consistently exposed to low levels of food contaminants (FCs) as a result of the repeated intake of everyday food [1,2,3]. These contaminants are prevalent even in healthy and sustainable diets, designed to meet nutritional needs, promote health and reduce the risk of chronic diseases while minimising environmental impacts. Yet there remains a limited understanding of their global concentrations and combinations across different dietary patterns [4]. The EAT-Lancet Commission Guidelines for healthy diets and sustainable food production systems recommend a high intake of plant-based foods (fruits, vegetables, legumes, whole grains, and nuts) and a moderate intake of animal-sourced foods [4]. The overarching principles are a balance of calories, adequacy of macronutrients and micronutrients, sustainability, and affordability. This guidance on healthy diets provides sufficient scope for different dietary patterns (e.g., omnivorous or vegetarian) to be considered for research purposes [4]. These guidelines are based primarily on food groups and formulated by combining evidence from epidemiological (observational and population-based studies), systematic reviews/meta-analyses, and randomised controlled trials (RCTs). Observational studies examine large populations over time and help identify long-term associations between dietary patterns (e.g., high consumption of plant-based foods and low intake of red meat) and the risk of chronic diseases like cardiovascular disease, cancer, and diabetes, establishing correlations rather than proving causation. The multi-faceted, holistic, and interdisciplinary approach that associates diet with health and environmental sustainability ensures that the recommendations are robust, actionable, and tailored for both human health and planetary well-being. However, although the authors considered it important, the issue of possible FCs, a global concern with varying magnitudes across different regions, was not addressed [4].

It is well known that exposure through dietary intake to a range of FCs, including pesticides, heavy metals, mycotoxins, heterocyclic amines (HAAs), and polycyclic aromatic hydrocarbons (PAHs), among others is unavoidable [5,6,7,8,9,10,11,12,13]. It is impractical to regulate every substance that could enter the food chain; therefore, the European Union prioritises maximum levels of FCs of the greatest potential to cause harm (Commission Regulation (EU) 2023/915) [14]. In 2023, the Rapid Alert System for Food and Feed (RASFF) recorded 4695 alerts, with pesticide residues being the most notified hazard category (936 notifications), and mycotoxins as the third most notified (401 notifications). Therefore, pesticides and mycotoxins are chemicals of special concern because they constitute the predominant border rejection notifications for food and feed in Europe but other chemical contaminant alerts include heavy metals and environmental pollutants [15]. Nevertheless, many FCs remain unregulated and therefore, unmonitored [16]. Human biomonitoring studies confirmed the co-occurrence of mycotoxins and pesticides in urine samples in European consumers [17,18].

The basic estimation of dietary exposure based on mg/kg body weight/day uses the average concentration of the contaminant in each food, the mean amount of the food consumed by the population, and the average body weight. More complex scenarios for estimating the real-life levels of mixtures of FCs require the integration of data from multiple sources and are a challenging issue for researchers, regulators, and industry [19]. In this context, OpenFoodTox provides crucial data on chemical risk in food and feed based on the assessment carried out by the European Food Safety Authority (EFSA) [20]. These data are crucial for gaining a better understanding of the impacts of chemicals on human health. In addition, platforms such as the Global Environment Monitoring System/Food (GEMS/Food) from the Codex Alimentarius Commission are valuable tools, providing detailed information [21] on the chemical composition of foods worldwide. These databases bring together a wide range of information to help assess and control food risks. Furthermore, the European Information Platform for Chemical Monitoring (https://ipchem.jrc.ec.europa.eu/, accessed on 30 September 2024) stands out as the main source of data on the occurrence of chemical substances in Europe, facilitating access to information on exposure and environmental pollution, essential for risk assessment studies [22]. Using data from the scientific literature published worldwide can be a reliable source of data for estimating exposure scenarios to FC mixtures if the data on the quantification of FCs in commonly consumed foods are systematically organised in a FAIR database. This strategy can enhance risk assessment frameworks and promote more accurate and protective regulations for food safety.

Testing mixtures is a toxicological research trend [23] and allows for the capture of complex interactions and better assess the cumulative risk of exposure to multiple contaminants, which is more reflective of potential health outcomes. Mixtures of FCs may interact in complex ways, potentially amplifying adverse health effects beyond those caused by single contaminants [24]. EFSA emphasised the importance of understanding how complex mixture exposure contributes to human disease because there is limited understanding of the real-life levels of exposure to multiple food contaminants (FCs) from different dietary choices. Studying the impact of mixtures of FCs by RCTs is nearly impossible, as these assays would require one group to be deliberately exposed to mixtures of contaminants (treatment group), while another group remains unexposed to the contaminants (control group) [24]. A promising approach to overcome this challenge is integrating the real-life levels of mixtures of FCs with physiologically relevant in vitro models to improve the understanding of how these mixtures behave in biological systems.

This study proposes a methodology to understand the mixtures of FCs that mimic different scenarios exposure, including real-life levels and worst-case scenarios, within omnivorous and vegetarian dietary models. The approach integrates the construction of a Findable, Accessible, Interoperable, and Reusable (FAIR) database, comprising bibliographic data on the occurrence of 73 FCs in 16 food items. It incorporates these data into omnivorous and vegetarian diets aligned with the EAT-Lancet Commission’s guidelines to calculate exposure from this dietary pattern’s consumption. It is essential to understand the exposure levels of FCs in real-life scenarios, in order to assess the potential risks associated with FC mixtures through in vitro studies. This improved understanding can help strengthen risk assessment frameworks and promote the establishment of more accurate and protective regulations for food safety, ensuring better protection of public health.

## 2. Materials and Methods

### 2.1. Construction of Dietary Models

Two dietary models were simulated following the EAT-Lancet Commission Reference Diet for Healthy and Sustainable Food Systems: one omnivorous and one vegetarian (Table 1). The macronutrient and micronutrient composition of these two models (Table S1) was estimated using the Portuguese Food Composition Table (TCAP), which provides the nutritional data for individual food items per 100 g, including both raw and cooked foods [25]. The nutritional information for each food item was retrieved and adjusted to the exact amounts used in each dietary model. The quantities of representative foods within each food group were selected to ensure that the energy value and macronutrient distribution were comparable between the two models. As a result, the total energy content was 2338 kcal for the omnivorous model and 2304 kcal for the vegetarian model, with protein, fat, and carbohydrate contents varying by less than 10% between the two models. In terms of macronutrient distribution, 42% of the total energy intake was derived from fat, 41% from carbohydrates, and 17% from protein. Both models are relatively balanced in micronutrients (Table S1); the vegetarian model, nonetheless, reflects the limited sources of vitamin D and B12 inherent to this dietary pattern, just as the omnivorous model demonstrates higher levels of calcium, potassium, and magnesium [26].

### 2.2. Compilation of the FAIR Database for Food Contaminants

A modified version of the FoodMine protocol (https://github.com/I3ALAQV/FoodMine, accessed on 30 June 2024) was used to conduct literature mining on PubMed concerning the occurrence (between 2000 and 2023) of 96 contaminants across different groups (heavy metals, polychlorinated biphenyls, dioxins, furans, polycyclic aromatic hydrocarbons (PAHs), pesticides, mycotoxins, and heterocyclic aromatic amines (HAAs)) in a wide variety of foods. For more information on the search strategy and machine learning protocol, please consult the Supplementary Materials of reference [27]. For the construction of a FAIR database, records of 16 widely consumed foods—4 animal-based (beef, chicken eggs, salmon, and cheese), and 12 plant-based (bread, pasta, rice, wheat, maize, potato, cabbage, carrot, apple, peanuts, olive oil)—were selected, totalling 151 records for data extraction. Of these, 6 were excluded based on specific criteria: records involving artificially contaminated food samples, food samples intended for animal feed, or studies using animals as experimental models. The remaining data, comprising over 1600 data points of the occurrence of the 73 contaminants (4 heavy metals, 18 PAHs, 10 pesticides, 29 mycotoxins, and 12 HAAs) in the selected foods, is available in the DIETxPOSOME FAIR database [28]. The database was carefully curated to ensure compliance with the FAIR principles, facilitating transparency and reproducibility. Relevant data were extracted from the text, tables, and figures or Supplementary Materials of the eligible records. The protocol followed for data extraction is available in Data S1. A total of 10 reviewers participated in data extraction, with each reviewer responsible for extracting data from 14 to 17 articles independently. Each reviewer then cross-checked the data extracted by another reviewer. After the data extraction and initial revision, two reviewers conducted a second review of the dataset and standardised the database.

### 2.3. Estimation of Food Contaminants Intake from Dietary Models

For each food and contaminant, global data from the FAIR database were utilised to derive a weighted estimate of mean values, as well as the 25th, 50th, and 75th percentiles. These percentiles capture the variability and distribution of contaminant levels across food items, providing a robust characterisation of potential exposure risks. The weighted estimation method adjusts for the relative frequency of data points from different studies, assigning greater weight to data from a larger sample size for a more reliable estimate. Median values were used as a secondary measure of central tendency when the mean was unavailable. Data points that were reported with only a minimum or maximum value and no associated mean or median values have been excluded to avoid biases or unreliable estimates. Data points from samples with undetected values (reported as being below the limits of detection and/or quantification) were included in the estimation as LOD/√2 or LOQ/√2 to mitigate the impact of censoring in the dataset. Before analysis, all the units were standardised to micrograms of contaminant per kilogram of food item.

Data reported on a dry weight basis were generally excluded from the derivation of estimates, except for the cereal group, which includes wheat, maize, and rice. Due to the limited availability of specific data on bread and pasta in the database, estimates for contaminant distribution in these food categories were derived from maize and wheat data, respectively. Regarding specific contaminants, enniatins A, B, A1, and B1 were summed to provide a single cumulative estimate for the presence of these compounds, labelled then as enniatins (ENNs). Similarly, individual ergot alkaloids (ergotamine, ergotaminine, ergokryptine, ergokryptinine, ergonisine, ergosine, ergometrine, ergocornine, and ergocristine) were aggregated and presented as ergot alkaloids (EgTs), and alternariol and alternariol methyl ether as alternaria toxins (ATs).

To map the daily contaminant burden in both diet models, three exposure scenarios were calculated based on the 25th, 50th, and 75th percentiles of the distribution of contaminant levels across food items. The total contribution of a contaminant for a given diet is given by the sum of the levels of contaminants across the food items of the diets according to their proportions in each diet.

### 2.4. Data Handling and Descriptive Statistics

Data analysis was conducted using Python v3.12.7 (https://www.python.org), NumPy v.2.1.1 (https://numpy.org), pandas v.2.2.2 (https://pandas.pydata.org), and Matplotlib v.3.9.2 (https://matplotlib.org). Descriptive statistical analyses were performed using SciPy v1.14.1 (https://scipy.org/) to ensure the accurate interpretation of data. Graphpad Prism v10.1.2 (Boston, MA, USA) was used for graphical visualisation.

## 3. Results and Discussion

**Figure 1:**
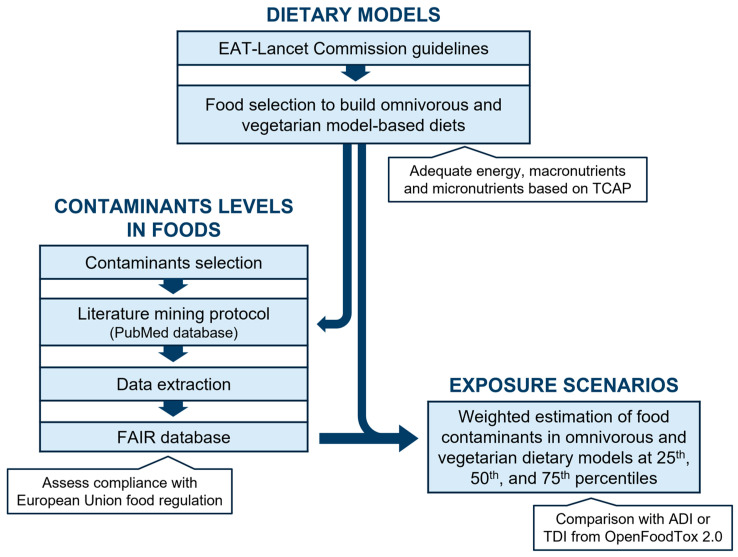
Outline of steps taken to estimate FC intake scenarios within two dietary models based on literature search. EAT-Lancet Commission Guidelines [4]; TCAP—Tabela de Composição de Alimentos Portuguesa [25]; ADI—acceptable daily intake; TDI—tolerable daily intake; OpenFoodTox 2.0 [20].

Data from the scientific literature containing the reliable quantification of FCs in foods were systematically organised, and the extracted data were incorporated into two distinct dietary models: omnivorous and vegetarian. To simulate various scenarios of FC mixtures and worst-case intakes, exposure levels at the 25th, 50th, and 75th percentiles were calculated for both models. These calculations integrated data from the dietary models and the compiled database following the scheme outlined in Figure 1.

The analysis of the FAIR database by itself enables a comprehensive discussion of the distribution of contaminants across different food groups. Furthermore, the integration of these data with dietary models provides exposure estimates for the two dietary patterns, which are discussed below. 

### 3.1. Contaminant Distribution in Animal- and Plant-Based Foods

**Figure 2:**
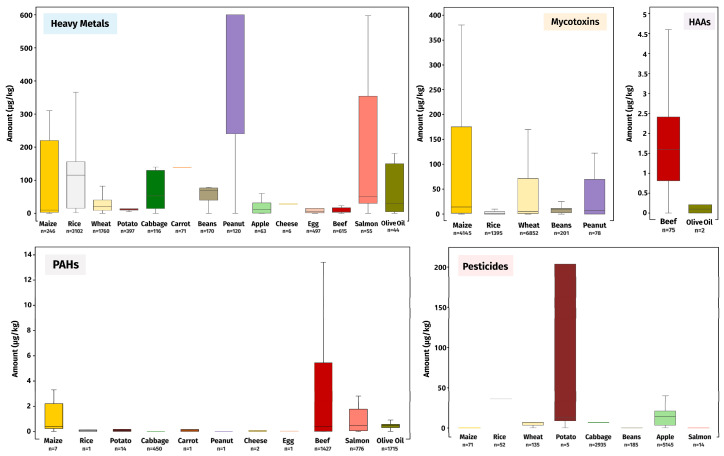
Distribution of contaminant levels across the food groups according to the FAIR database [23]. Boxplots were organised by the distribution of the weighted average of the contaminants within each group across the 14 selected foods.

Heavy metals, pesticides, and mycotoxins are the most studied groups among the selected FCs, with 7262, 8542, and 12,671 data points, respectively. The distribution of heavy metals was found to occur across both animal- and plant-based foods, but higher levels appear in maize, rice, peanuts, and salmon. Pesticides, in turn, with 8542 data points, were found mainly in plant-based items, with higher amounts found in potatoes and apples, although residues were also reported in salmon. Mycotoxins are found in higher amounts in maize, wheat, and peanuts and lower levels in rice and beans. Furthermore, HAAs (77 data points) were only reported in beef and olive oil, while PAHs (4395 data points) were distributed in both plant (except wheat, beans, and apple) and animal items (Figure 2).

**Figure 3:**
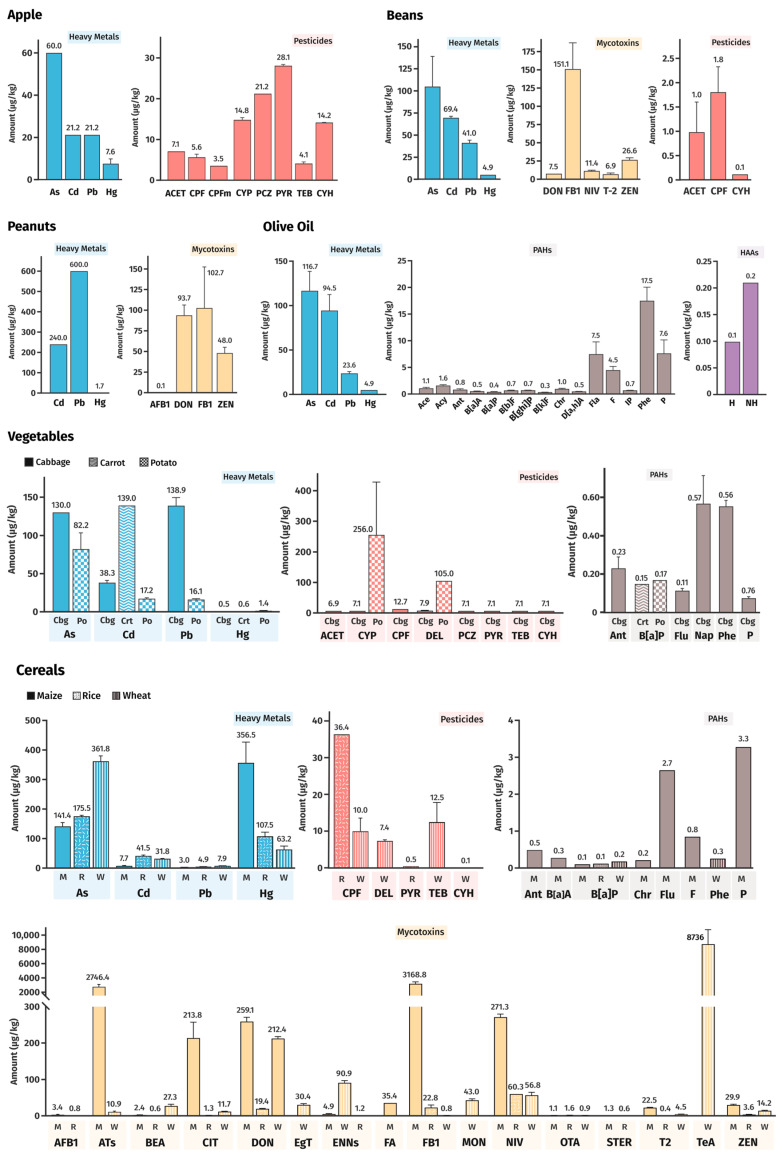
Identification and weighted mean value of FCs in each plant-based food. Ats, alternaria toxins; Ace, Acenaphthene; ACET, acetamiprid; Acy, Acenaphthylene; AFB1, aflatoxin B1; Ant, Anthracene; BEA, Beauvericin; B[a]A, Benz[a]anthracene; B[b]F, Benzo[b]fluoranthene; B[k]F, Benzo[k]fluoranthene; B[a]P, Benzo[a]pyrene; B[ghi]P, Benzo[g,h,i]perylene; Cbg, cabbage; Crt, carrot; Chr, Chrysene; CIT, Citrinin; CPF, chlorpyrifos; CPFm, chlorpyrifos-methyl; CYH, λ-cyhalothrin; CYP, cypermethrin; D[ah]A, Dibenz[a,h]anthracene; DEL, deltamethrin; DON, deoxynivalenol; ENNs, enniatins; EgT, ergot alkaloids; FA, Fusaric Acid; FB1, fumonisin B1; Fla, fluoranthene; F, Fluorene; Flu, fluoranthene; H, harman; HAAs, heterocyclic aromatic amines; IP, Indeno[1,2,3-cd]pyrene; PAHs, polycyclic aromatic hydrocarbons; Phe, Phenanthrene; Po, potato; P, pyrene; M, maize; MON, Moniliformin; Nap, Naphthalene; NH, norharman; NIV, nivalenol; OTA, ochratoxin A; PCZ, propiconazole; PYR, pyraclostrobin; R, rice; STER, Sterigmatocystin; TEB, tebuconazole; T2, T-2 toxin; TeA, Tenuazonic acid; ZEN, zearalenone; W, wheat.

The 14 foods evaluated presented a remarkably different profile of FCs. The distribution of FCs in the selected plant foods was as follows, as depicted in Figure 3:

- Apples: metals and pesticides;
- Beans: metals, mycotoxins, and pesticides;
- Peanuts: metals and mycotoxins;
- Olive oil: metals, PAHs, harman, and norharman;
- Vegetables: metals, pesticides, and PAHs;
- Cereals: metals, pesticides, PAHs, and mycotoxins.

Metals were present in all the plant food items, making them the most ubiquitous contaminant reported. Pesticides were found in four out of six groups and mycotoxins and PAHs in half of the groups studied. European Union (EU) legislation sets maximum allowable limits for certain contaminants in specific foods; however, only a restricted number of molecules are covered. Consequently, the safety assessment of such foods in relation to nonregulated contaminants is hindered since not all contaminant levels effectively present in food can be framed concerning regulatory levels. For the ones considered in legislation, however, in general, the weighted mean value of data present in Figure 3 was below the maximum levels allowed in these foods (Commission Regulation (EU) 2023/915) [14], although there were some exceptions, namely, cadmium in peanuts, apple, beans, and carrot; arsenic in rice and lead; and fumonisin B1 and aflatoxin B1 in maize. Concerning pesticide residues, some exceed the maximum residue levels in the EU [29], namely, chlorpyrifos-methyl in rice, propiconazole in apples, and chlorpyrifos and cypermethrin in potatoes. However, it is important to note that the FAIR database is based on retrospective data (from scientific publications between 2000 and 2023), and the maximum permitted contaminant levels have been recently updated. As a result, it is understandable that the average levels found in some foods may exceed the current legal limits.

**Figure 4:**
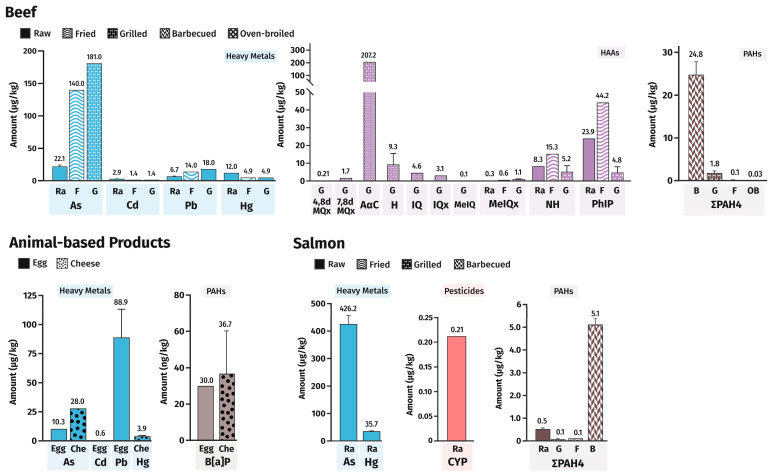
Identification and weighted mean value of FCs in each animal-based food. For salmon and beef, Ra means raw, G means grilled, F means fried which encompasses fried and pan-fried samples, B means barbecued, while OB means oven-broiled and refers to roasting and oven-broiled samples. 4,8dMQx, 2-amino-3,4,8-trimethylimdazo[4,5-f]quinoxaline; 7,8dMQx, 2-Amino-3,7,8-trimethylimidazo(4,5-f)quinoxaline; AαC, 2-amino-α-carboline; B[a]A, Benz[a]anthracene; B[a]P, Benzo[a]pyrene; B[b]F, Benzo[b]fluoranthene; Che, cheese; CYP, cypermethrin; Chr, Chrysene; H, harman; IQ, 2-Amino-3-methyl-3H-imidazo[4,5-f]quinoline; IQx, 3-Methyl-3H-imidazo[4,5-f]quinoxalin-2-amine; MeIQ, 2-amino-3,4-dimethylimdazo[4,5-f]quinoline; MeIQx, 2-amino-3,8-dimethylimdazo[4,5-f]quinoxaline; NH, norharman; PhIP, 2-amino-1-methyl-6-phenylimidazo[4,5-b]pyridine. PAH4 relates to the sum of B[a]P, B[a]A, Chr, and B[b]F selected by EFSA based on their carcinogenic potential and frequency of occurrence in meats; for comprehensive data on all PAHs, see Supplementary Materials Table S2.

FC distribution in selected animal-based foods was as follows (Figure 4):

- Eggs: metals and benzo[a]pyrene;
- Cheese: metals and benzo[a]pyrene;
- Salmon: metals, cypermethrin, and PAHs;
- Beef: metals, HAAs, and PAHs.

As with plant foods, metals were distributed across all the food groups. In animal-based foods, the same was true for PAHs. Pesticides were only reported for salmon and no mycotoxins were found in animal foods. Concerning regulated FCs, the weighted mean value of PAHs was below the maximum levels for meat and meat products [14]. More information concerning PAHs in raw and cooked beef and salmon is detailed in Table S2. HAAs, IQ, PhIP, MeIQ, and MeIQx are mainly formed in the crust or gravy by the heating of muscle foods [6]. Temperature and cooking time, the presence of precursors such as creatine, reducing sugars and free amino acids, and cooking methods, such as marinating, influence the formation of HAAs [6,30]. These are examples of FCs that are not regulated in the EU, although IQ is classified by IARC as probably carcinogenic to humans (Group 2A), while PhIP, MeIQ, and MeIQx are classified as possibly carcinogenic to humans (Group 2B), which highlights the importance of considering them in food safety evaluation and the in vitro toxicological evaluation of FC mixtures.

### 3.2. Contaminant Distribution in Omnivorous Versus Vegetarian Dietary Models

**Figure 5:**
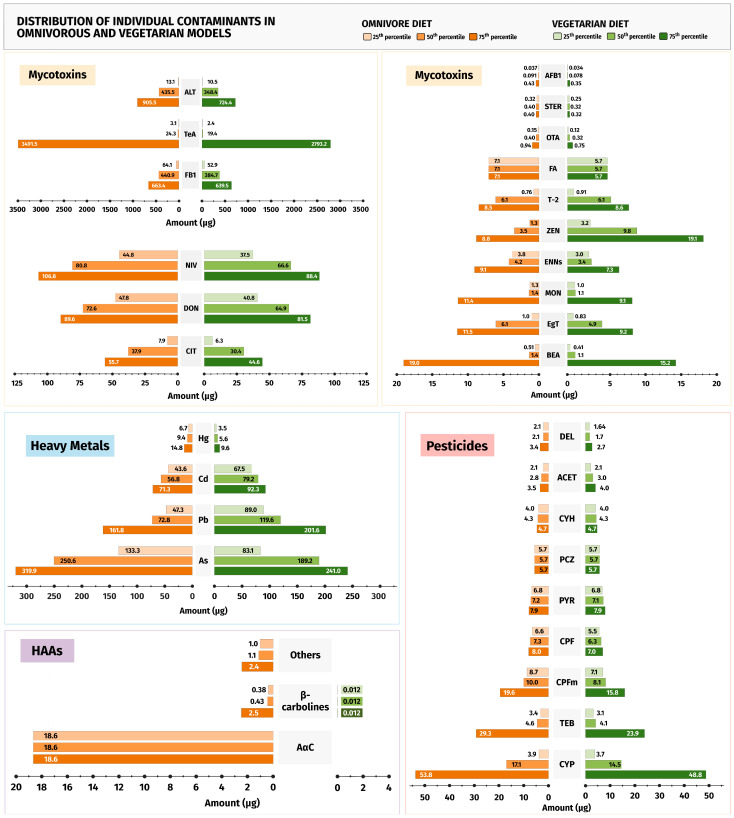

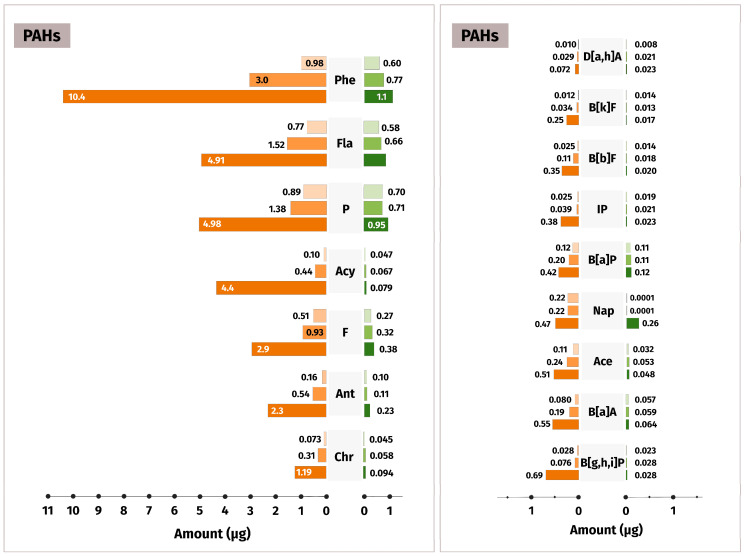
Mirror plots of the distribution of individual contaminants in the omnivorous and vegetarian models at the 25th, 50th, and 75th percentile scenarios, organised by group of contaminants. Ace, Acenaphthene; ACET, acetamiprid; Acy, Acenaphthylene; AFB1, aflatoxin B1; Ant, Anthracene; ATs, alternaria toxins; BEA, Beauvericin; B[a]A, Benz[a]anthracene; B[a]P, Benzo[a]pyrene; B[b]F, Benzo[b]fluoranthene; B[k]F, Benzo[k]fluoranthene; B[ghi]P, Benzo[g,h,i]perylene; CIT, Citrinin; Chr, Chrysene; CPF, chlorpyrifos; CPFm, chlorpyrifos-methyl; CYH, λ-cyhalothrin; CYP, cypermethrin; D[ah]A, Dibenz[a,h]anthracene; DEL, deltamethrin; 4,8dMQx, 2-amino-3,4,8-trimethylimdazo[4,5-f]quinoxaline; 7,8dMQx, 2-Amino-3,7,8-trimethylimidazo(4,5-f)quinoxaline; DON, deoxynivalenol; ENNs, enniatins; EgT, ergot alkaloids; FA, Fusaric Acid; FB1, fumonisin B1; F, Fluorene; Fla, fluoranthene; H, harman; HAAs, heterocyclic aromatic amines; IP, Indeno[1,2,3-cd]pyrene; MON, Moniliformin; Nap, Naphthalene; NIV, nivalenol; NH, norharman; OTA, ochratoxin A; P, pyrene; PAHs, polycyclic aromatic hydrocarbons; PCZ, propiconazole; Phe, Phenanthrene; PYR, pyraclostrobin; STER, Sterigmatocystin; T2, T-2 toxin; TeA, Tenuazonic acid; TEB, tebuconazole; ZEN, zearalenone. For HAAs, the results were grouped in β-carbolines (sum of H and NH) and others include 4,8-diMeIQx, 7,8-diMeIQx, IQ, IQx, MeIQx, MeIQ, and PhIP (individual data are available in Table S3).

A weighted estimate of the 25th, 50th, and 75th percentiles of both the omnivorous and vegetarian models was assessed from the FAIR database and summarised in Table 2 by group of FCs, and in Figure 5 by individual contaminants. The weighting process adjusts for variations in data availability across the scientific literature, allowing us to generate more accurate and representative percentiles for each contaminant in the selected foods used to build the dietary models. Globally, the omnivorous model presents slightly higher levels of FCs, potentially due to the adjustments of food amounts to have a comparable balance of nutrients, since plant-based foods were the same in both diets (Table 2). The exceptions are the higher intake of PAHs, particularly heavy PAHs, and the heterocyclic amines PhIP, 8-MeIQx, and 7,8-MeIQx in the omnivorous model compared to the vegetarian, as detailed in Figure 5. These differences are explained because the levels of PAHs from plant foods are usually far less than those from meat and fish cooked at high temperatures or smoked, and HAAs are primarily associated with animal proteins and therefore, not typically found in plant foods [30,31,32].

Different countries have different limits for chemical contaminants in food [10]. This lack of harmonisation of contaminant limits causes differences in food safety standards across borders. The European Union’s model of food safety in relation to chemical contaminants in food is considered a point of reference by the World Health Organization [33]. Therefore, to assess whether the intake levels of contaminants are within safe limits, based on toxicological data, our intake data were compared with a comprehensive and reliable source, the EFSA chemical hazard database. A detailed comparison was thus made with the estimated contaminants in both model diets at the 25th and 75th percentiles with data from OpenFoodTox2.0 [20].

For cadmium and mercury, EFSA established tolerable weekly intakes (TWIs) of 2.5 and 4 µg/kg bw, respectively, which corresponds to 175 and 280 µg/week for an average adult weighing 70 kg, translating to daily intakes of 25 µg and 16 µg, respectively. Only the mercury levels in both dietary models are within safe limits for the 25th and 75th percentiles. In contrast, the estimated levels of cadmium exceed the TWI.

Concerning inorganic arsenic and lead, EFSA concluded that health-based guidance values, such as TWI, are no longer appropriate due to their inherent toxicity. Consequently, for inorganic arsenic, EFSA established a benchmark dose lower confidence limit (BMDL_05_) of 0.06 µg/kg bw/day, which translates to approximately 4.2 µg/day for a 70 kg adult, related to skin cancer risk [34]. However, the EFSA CONTAM Panel decided not to determine a value for an MOE (Margin of Exposure) of low concern; thus, an MOE of one was used. For lead, the BMDL_01_ values were set at 0.5 and 1.5 µg/kg bw/day for developmental neurotoxicity and systolic blood pressure, respectively, and BMDL_10_ was set at 0.63 µg/kg bw/day for nephrotoxicity [35]. These values correspond to approximately 35 µg/day, 105 µg/day, and 44.1 µg/day for a 70 kg individual. The comparison between the estimated levels of arsenic and lead in both dietary models at the 25th and 75th percentiles exceed the BMDL established by EFSA, suggesting a potential risk if sustained over time. The exception was the 25th percentile for systolic blood pressure.

Regarding pesticides, the acceptable daily intake (ADI) based on the EFSA guidelines is 0.025; 0.005; 0.01; 0.001; 0.02; 0.07; 0.03; 0.03; and 0.005 mg/kg body weight/day, respectively, for acetamiprid, cypermethrin, deltamethrin, chlorpyrifos, methomyl, propiconazole, pyraclostrobin, tebuconazole, and λ-cyhalothrin [20]. For an average adult, this corresponds to 1.75; 0.35; 0.7; 0.07; 1.4; 4.9; 2.1; 2.1; and 0.35 mg/day, respectively. The estimated intake level of those residues of pesticides on both diet models at the 25th and 75th percentiles is lower than the EFSA acceptable daily intakes, notwithstanding the EU lowered the maximum residue limits (MRLs) for pesticides in recent years and mining is based on retrospective data.

Concerning mycotoxins, the tolerable daily intake based on EFSA guidelines is 1; 0.1; 1.2; 0.1; and 0.25 µg/kg body weight/day, respectively, for deoxynivalenol, fumonisin B1, nivalenol, T-toxin, and zearalenone [20]. For an average adult (weighing 70 kg), this corresponds to 70; 7; 84; 7; 17.5; and 331–1015 µg/day, respectively, for deoxynivalenol, fumonisin B1, nivalenol, T-toxin, zearalenone, and ochratoxin A. The estimated exposure to those mycotoxins on both dietary models at the 25th percentile would likely fall within safe limits for an average adult, except for fumonisin B1. For the 75th percentile, the estimated exposure was slightly higher than the reference levels for deoxynivalenol, nivalenol, T-toxin, and zearalenone, and very high for fumonisin B1. This could be explained because the literature mining is based on data from all around the world and some countries have heavy contamination of mycotoxins. For ochratoxin A, the TWI established in 2006 is no longer valid; thus, an MOE of low health concern was set at 200 for non-neoplastic effects and 10,000 for neoplastic effects. The BMDL_10_ of 4.73 and 14.5 µg/kg body weight/day for non-neoplastic and neoplastic effects, respectively, was established [36]. The MOE for non-neoplastic effects was above 200 for a worst-case scenario, whereas the MOE for neoplastic effects was below 10,000 even at the 25th percentile, which constitutes a possible health concern. Moreover, no limits were established for the other studied mycotoxins.

According to EFSA Scientific Opinion of the Panel on Contaminants in the Food Chain concerning PAHs in Food, average dietary exposure across European countries for which data are available is 0.235 µg/day for benzo[a]pyrene (range: 0.185–0.255 µg/day) and 1.729 µg/day for PAH8 (sum of B[a]P; Chr; B[a]A; B[b]F; B[k]F; D[ah]A; B[ghi]P; and IP) (range: 1.415–2.136 µg/day) [37]. The estimated intake level of those PAHs in the omnivorous model at the 25th percentile and at the 50th percentile would likely fall within safe limits, whereas the 75th percentile was slightly above the range found in this study from 2008 [34]. Organisations such as the EFSA and other regulatory bodies use PAH8 levels as indicators to ensure food safety, especially in processed, smoked, or grilled foods, as these cooking methods can increase PAH levels in foods [38].

In the EU, there is a lack of data concerning HAA exposure, as recently highlighted by the Panel on Contaminants of the Norwegian Scientific Committee for Food and Environment, which summarised the knowledge on the formation of several carcinogenic process contaminants in grilled food [37]. The assessment of mean total dietary exposure to two HAAs based on the consumption of meats and breads in the United States population was estimated to be 0.565 µg/day, with 0.473 µg/day from PhIP, and 0.0917 µg/day from MeIQx [39]. The estimated exposure scenario of those HAAs in the omnivorous model at the 25th percentile would likely fall within safe limits, whereas the 75th percentile was above the range found in this study from 2018. 

The proposed approach for estimating FC exposure scenarios across different dietary models has several considerations. The quality of the data is influenced by the original studies being mined, with potential biases introduced by variations in methodologies, geographic regions, sample sizes, and study designs. These factors could affect the reliability of the associations drawn. Additionally, the application of this methodology may be limited in cases where the scientific literature is sparse, as is the case with some emerging contaminants. Additionally, certain minor dietary constituents, such as spices, herbs, coffee, tea, and salt, which may contain additional FCs, were not included. Nevertheless, the estimated FCs in both dietary models at the 25th percentile likely reflect the real-world exposure levels, as most contaminants would fall within the EFSA reference limits, with a few exceptions (e.g., arsenic, cadmium, lead for developmental neurotoxicity and nephrotoxicity, and fumonisin B1). Moreover, at the 75th percentile, these limits were exceeded for those FCs, along with some additional mycotoxins, representing a worst-case scenario that slightly exceeds the reference values for metals and mycotoxins. Using in vitro models, the 25th percentile can mimic real-life exposure to FCs, while the 75th percentile simulates a possible worst-case scenario, and these percentiles can be used to better understand the burden of foodborne disease through associations with adverse health outcomes [40].

## 4. Conclusions

A systematic literature mining approach was employed to characterise the occurrence of FCs across specific foods to identify trends in their distribution, and to search for meaningful associations between two different dietary models and prevalent FCs. Moreover, exposure scenarios using different levels of contamination were successfully assessed. By literature mining, it becomes easier to explore, create connections, and uncover new patterns that would otherwise go unnoticed. In the future, the code can be adapted to other search engines (e.g., Scopus and Web of Science), which have different criteria for indexing and retrieving literature based on search terms, and the use of multiple APIs will widen search. 

The data collected was used to build a FAIR database, published in Zenodo, an open-access repository developed under the European OpenAIRE program for the deposition of datasets (http://zenodo.org, accessed on 30 June 2024), which allows the scientific community to access and use the data for future studies and to complement it with a higher number of foods or FCs.

A weighted estimate of the 25th, 50th, and 75th percentiles of FCs in both dietary models revealed that the omnivorous dietary model exhibited a higher intake of PAHs, particularly heavy PAHs, and HAAs, namely, PhIP, 8-MeIQx, and 7,8-MeIQx, compared to the vegetarian model. Regarding the other contaminants, only marginal increases in FC levels were observed in the omnivorous model in comparison to the vegetarian. The At 25th percentile, the FC levels in both dietary models fall within the European Food Safety Authority (EFSA) reference exposure levels for chemical hazards, except for arsenic, lead, cadmium, fumonisin B1, and OTA. At the 75th percentile, the FC levels exceed the EFSA reference levels for those FCs and additional mycotoxins. Using in vitro models, the 25th percentile can mimic real-life FC exposure, while the 75th percentile simulates a possible worst-case scenario. The comparison between the estimated scenarios of FC exposure in both dietary models at the 25th percentile and the EFSA chemical hazard database reveals that at this percentile, the levels of FCs fall within reference limits, except for arsenic, lead, cadmium, fumonisin B1, and OTA for neoplastic effects. The 75th percentile was above those levels for a higher number of FCs, which can be interpreted as a worst-case scenario. This information is important to mimic complex FC exposure scenarios and develop an experimental mixture to be used in in vitro assays simulating the real-life levels of lower contaminant exposure and worst-case scenarios to prospect for potential risks in the context of food and health.

Using mixtures of FCs in in vitro toxicological assays, instead of testing single compounds, although challenging, is becoming increasingly important for several reasons related to the complexity of real-world exposures, the combined effects of multiple substances, and the need to improve the risk assessment and the toxicity data for mixtures. The proposed methodology, based on a FAIR database, can be customised to be used in future research with different dietary models or different contaminants whose occurrence data can only be found in bibliographic records, and not yet reported in curated databases.

## Figures and Tables

**Table 1:** Comparison of food quantities and energetic contributions across omnivorous and vegetarian dietary models according to reference diet [4]. Estimation was based on TCAP data [25].

	Reference Diet			Omnivorous Diet		Vegetarian Diet	
Food Group	g/Day	kcal/Day	Food	g/Day	kcal/Day	g/Day	kcal/Day
Whole grains	200–600	811		600	830	480	664
			Rice	200	250	160	200
			Wheat	200	204	160	163
			Maize	200	376	160	301
Tubers/Starchy vegetables	0–100	39	Potato	50	44	40	35
Vegetables	200–600	78		350	82	350	82
			Cabbage	200	44	200	44
			Carrot	150	38	150	38
Fruits	100–300	126	Apple	200	122	200	122
Dairy foods	0–500	153	Cheese	60	148	70	173
Animal protein sources	0–211	151		210	500	120	179
			Beef	90	173	-	-
			Egg	-	-	120	179
			Salmon	120	328	-	-
Other protein sources	25–250	575		60	162	280	690
			Beans	40	41	200	206
			Peanuts	20	121	80	484
Added fats	20–91.8	450	Olive oil	50	450	40	360
Added sugars	0–31	120	-	-	-	-	-
Total	-	2503		1580	2338	1580	2304

**Table 2:** Contaminant burden per chemical group in different exposure scenarios (25th, 50th, and 75th percentiles) in omnivorous and vegetarian models (detailed in Table 1).

Diet	Percentile	Heavy Metals (µg)	HAAs (µg)	Mycotoxins (µg)	Pesticides (µg)	PAHs (µg)	Global (µg)	Global (µg/kg)
Omnivorous	25th	230.9	20.1	196.9	43.2	4.11	495.1	313.4
	50th	389.7	20.2	1122	61	9.29	1602	1014
	75th	567.9	23.6	5389	135.8	34.76	6152	3893
Vegetarian	25th	243.1	0.01	165.9	39.7	2.6	451.3	285.7
	50th	393.6	0.01	947	54.7	3	1398	885.1
	75th	544.4	0.01	4447	120.5	4.33	5116	3238

## Data Availability

The original data presented in the study are openly available in Zenodo at https://zenodo.org/records/12584594.

## Supplementary Materials

The following supporting information can be downloaded at https://www.mdpi.com/article/10.3390/foods13223659/s1, Table S1: Nutritional composition of omnivorous and vegetarian dietary models, estimated based on TCAP data [25].; Data S1: Details and protocol for the construction of the DIETxPOSOME FAIR database [28].; Table S2: Detailed weighted mean values of PAHs for beef and salmon across different cooking types.; Table S3: Distribution of individual HAAs in omnivorous (OMN) and vegetarian (VEG) models at the 25th, 50th, and 75th percentile scenarios.

## Abbreviations

4:8dMQx, 2-amino-3,4,8-trimethylimdazo[4,5-f]quinoxaline; 7,8dMQx, 2-Amino-3,7,8-trimethylimidazo(4,5-f)quinoxaline; AαC, 2-amino-α-carboline; ATs, alternaria toxins; Ace, Acenaphthene; ACET, acetamiprid; Acy, Acenaphthylene; AFB1, aflatoxin B1; Ant, Anthracene; B[a]A, Benz[a]anthracene; B[a]P, Benzo[a]pyrene; B[b]F, Benzo[b]fluoranthene; BEA, Beauvericin; B[ghi]P, Benzo[g,h,i]perylene; B[k]F, Benzo[k]fluoranthene; Chr, Chrysene; CIT, Citrinin; CYP, cypermethrin; CPF, chlorpyrifos; CPFm, chlorpyrifos-methyl; D[ah]A, Dibenz[a,h]anthracene; DEL, deltamethrin; DON, deoxynivalenol; ENNs, enniatins; EgT, ergot alkaloids; FB1, fumonisin B1; Fla, fluoranthene; F, Fluorene; FA, Fusaric Acid; H, harman; HAAs, heterocyclic aromatic amines; IP, Indeno[1,2,3-cd]pyrene; IQ, 2-Amino-3-methyl-3H-imidazo[4,5-f]quinoline; IQx, 3-Methyl-3H-imidazo[4,5-f]quinoxalin-2-amine; CYH, λ-cyhalothrin, MeIQ, 2-amino-3,4-dimethylimdazo[4,5-f]quinoline; MeIQx, 2-amino-3,8-dimethylimdazo[4,5-f]quinoxaline; MON, Moniliformin; Nap, Naphthalene; NH, norharman; NIV, nivalenol; OTA, ochratoxin A; PAHs, polycyclic aromatic hydrocarbons; PCZ, propiconazole; Phe, Phenanthrene; PhIP, 2-amino-1-methyl-6-phenylimidazo[4,5-b]pyridine; P, pyrene; PYR, pyraclostrobin; STER, Sterigmatocystin; T2, T-2 toxin; TeA, Tenuazonic acid; TEB, tebuconazole; ZEN, zearalenone.

## References

[1] Eskola M., Elliott C.T., Hajšlová J., Steiner D., Krska R. (2020). Towards a dietary-exposome assessment of chemicals in food: An update on the chronic health risks for the European consumer. Crit. Rev. Food Sci. Nutr..

[2] Eskola M., Kos G., Elliott C.T., Hajšlová J., Mayar S., Krska R. (2020). Worldwide contamination of food-crops with mycotoxins: Validity of the widely cited ‘FAO estimate’ of 25%. Crit. Rev. Food Sci. Nutr..

[3] Stiefel C., Stintzing F. (2023). Endocrine-active and endocrine-disrupting compounds in food—Occurrence, formation and relevance. NFS J..

[4] Willett W., Rockström J., Loken B., Springmann M., Lang T., Vermeulen S., Garnett T., Tilman D., DeClerck F., Wood A. (2019). Food in the Anthropocene: The EAT–Lancet Commission on healthy diets from sustainable food systems. Lancet.

[5] Qu Z., Ren X., Du Z., Hou J., Li Y., Yao Y., An Y. (2024). Fusarium mycotoxins: The major food contaminants. mLife.

[6] Cordeiro T., Viegas O., Silva M., Martins Z.E., Fernandes I., Ferreira I., Pinho O., Mateus N., Calhau C. (2020). Inhibitory effect of vinegars on the formation of polycyclic aromatic hydrocarbons in charcoal-grilled pork. Meat Sci..

[7] Melo A., Cunha S.C., Mansilha C., Aguiar A., Pinho O., Ferreira I.M. (2012). Monitoring pesticide residues in greenhouse tomato by combining acetonitrile-based extraction with dispersive liquid-liquid microextraction followed by gas-chromatography-mass spectrometry. Food Chem..

[8] Pinto E., Almeida A., Ferreira I.M. (2016). Essential and non-essential/toxic elements in rice available in the Portuguese and Spanish markets. J. Food Compos. Anal..

[9] Thompson L.A., Darwish W.S. (2019). Environmental Chemical Contaminants in Food: Review of a Global Problem. J. Toxicol..

[10] Onyeaka H., Ghosh S., Obileke K., Miri T., Odeyemi O.A., Nwaiwu O., Tamasiga P. (2024). Preventing chemical contaminants in food: Challenges and prospects for safe and sustainable food production. Food Control.

[11] Rather I.A., Koh W.Y., Paek W.K., Lim J. (2017). The Sources of Chemical Contaminants in Food and Their Health Implications. Front. Pharmacol..

[12] Ray S., Vashishth R. (2024). From water to plate: Reviewing the bioaccumulation of heavy metals in fish and unraveling human health risks in the food chain. Emerg. Contam..

[13] Peloso M., Minkoumba Sonfack G., Prizio I., Baraldini Molgora E., Pedretti G., Fedrizzi G., Caprai E. (2024). Climate Effects on Ergot and Ergot Alkaloids Occurrence in Italian Wheat. Foods.

[14] European Commission (2023). Commission Regulation (EU) 2023/915 of 25 April 2023 on Maximum Levels for Certain Contaminants in Food and Repealing Regulation (EC) No 1881/2006. OJEU.

[15] European Commission 2023 Annual Report Alert and Cooperation Network. https://food.ec.europa.eu/document/download/911d49f2-b3ef-4752-8ea3-5f20dbbe9945_en?filename=acn_annual-report_2023.pdf.

[16] van der Fels-Klerx H.J., van Asselt E.D., van Leeuwen S.P.J., Dorgelo F.O., Hoek-van den Hil E.F. (2024). Prioritization of chemical food safety hazards in the European feed supply chain. Compr. Rev. Food Sci. Food Saf..

[17] Heyndrickx E., Sioen I., Huybrechts B., Callebaut A., De Henauw S., De Saeger S. (2015). Human biomonitoring of multiple mycotoxins in the Belgian population: Results of the BIOMYCO study. Environ. Int..

[18] Gallardo-Ramos J.A., Marín-Sáez J., Sanchis V., Gámiz-Gracia L., García-Campaña A.M., Hernández-Mesa M., Cano-Sancho G. (2024). Simultaneous detection of mycotoxins and pesticides in human urine samples: A 24-h diet intervention study comparing conventional and organic diets in Spain. Food Chem. Toxicol..

[19] Udovicki B., Djekic I. (2024). Quick Roadmap for Exposure Assessment of Contaminants in Food. Standards.

[20] Benfenati E., Roncaglioni A., Carnesecchi E., Mazzucotelli M., Marzo M., Toropov A., Toropova A., Baldin R., Ciacci A., Kovarich S. (2021). Maintenance, update and further development of EFSA’s Chemical Hazards: OpenFoodTox 2.0. EFSA Support. Publ..

[21] Gwynne M.D. (1982). The Global Environment Monitoring System (GEMS) of UNEP. Environ. Conserv..

[22] Comero S., Dalla Costa S., Cusinato A., Korytar P., Kephalopoulos S., Bopp S., Gawlik B.M. (2020). A conceptual data quality framework for IPCHEM—The European Commission Information Platform for chemical monitoring. TrAC Trends Anal. Chem..

[23] Di Piazza G., Dujardin B., Levorato S., Medina P., Mohimont L., Solazzo E., Costanzo V., European Food Safety Authority (EFSA) (2024). Prioritisation of pesticides and target organ systems for dietary cumulative risk assessment based on the 2019–2021 monitoring cycle. EFSA J..

[24] Mustafa E., Valente M.J., Vinggaard A.M. (2023). Complex chemical mixtures: Approaches for assessing adverse human health effects. Curr. Opin. Toxicol..

[25] Instituto Nacional de Saúde Doutor Ricardo Jorge (2023). Tabela de Composição de Alimentos Portuguesa—Version 6.0. https://portfir-insa.min-saude.pt/.

[26] EFSA Dietary Reference Values for the EU. https://www.efsa.europa.eu/en/topics/topic/dietary-reference-values.

[27] Martins Z.E., Ramos H., Araújo A.M., Silva M., Ribeiro M., Melo A., Mansilha C., Viegas O., Faria M.A., Ferreira I.M. (2023). From data to insight: Exploring contaminants in different food groups with literature mining and machine learning techniques. Curr. Res. Food Sci..

[28] Ramos H., Reis-Mendes A., Martins Z.E., Borges C.B., Araujo A.M., Silva M., Ribeiro M., Viegas O., Melo A., Faria M. (2024). DIETxPOSOME: A FAIR Database Detailing Food Contaminants Occurrence in Selected Foods. https://zenodo.org/records/12584594.

[29] EU Pesticides Database (v3.2). https://ec.europa.eu/food/plant/pesticides/eu-pesticides-database/start/screen/mrls.

[30] Costa M., Viegas O., Melo A., Petisca C., Pinho O., Ferreira I.M. (2009). Heterocyclic aromatic amine formation in barbecued sardines (Sardina pilchardus) and Atlantic salmon (*Salmo salar*). J. Agric. Food Chem..

[31] Savin R.-L., Ladoși D., Ladoși I., Păpuc T., Becze A., Cadar O., Torök I., Simedru D., Mariș Ș.C., Coroian A. (2024). Influence of Fish Species and Wood Type on Polycyclic Aromatic Hydrocarbons Contamination in Smoked Fish Meat. Foods.

[32] Vignesh A., Amal T.C., Vasanth K. (2024). Food contaminants: Impact of food processing, challenges and mitigation strategies for food security. Food Res. Int..

[33] European Court of Auditors (2019). Chemical Hazards in Our Food: EU Food Safety Policy Protects Us but Faces Challenges. European Union.

[34] Schrenk D., Bignami M., Bodin L., Chipman J.K., del Mazo J., Grasl-Kraupp B., Hogstrand C., Hoogenboom L., Leblanc J.-C., EFSA Panel on Contaminants in the Food Chain (CONTAM) (2024). Update of the risk assessment of inorganic arsenic in food. EFSA J..

[35] EFSA Panel on Contaminants in the Food Chain (CONTAM) (2010). Scientific Opinion on Lead in Food. EFSA J..

[36] Schrenk D., Bodin L., Chipman J.K., del Mazo J., Grasl-Kraupp B., Hogstrand C., Hoogenboom L., Leblanc J.-C., Nebbia C.S., EFSA Panel on Contaminants in the Food Chain (CONTAM) (2020). Risk assessment of ochratoxin A in food. EFSA J..

[37] EFSA Panel on Contaminants in the Food Chain (CONTAM) (2008). Polycyclic Aromatic Hydrocarbons in Food—Scientific Opinion of the Panel on Contaminants in the Food Chain. EFSA J..

[38] Mariussen E., Alexander J., Bukhvalova B.A., Dahl L., Olsen A.-K.H., Kvalem H.E., Schlabach M., Amlund H., Hannisdal R., Ruus A. (2024). Risk assessment of grilled and barbecued food. Food Risk Assess Eur..

[39] Pouzou J.G., Costard S., Zagmutt F.J. (2018). Probabilistic assessment of dietary exposure to heterocyclic amines and polycyclic aromatic hydrocarbons from consumption of meats and breads in the United States. Food Chem. Toxicol..

[40] Choudhury S., Medina-Lara A., Smith R., Daniel N. (2022). Research on health impacts of chemical contaminants in food. Bull. World Health Organ..

