# Supplementary material for: Exposure Scenarios for Estimating Contaminant Levels in Healthy Sustainable Dietary Models: Omnivorous vs. Vegetarian
Source: Foods. 2024 Nov 17;13(22):3659. doi: 10.3390/foods13223659 (PMC11593607; doi:10.3390/foods13223659)
Supplement: Supplementary file 1 [file foods-13-03659-s001.zip › Data S1.pdf]

Data S1: Details and protocol for the construction of DIETxPOSOME FAIR database [28].

Selection Criteria

Inclusion Criteria: Records pertaining to the occurrence of chemical contaminants (see table below for list of accepted contaminants) in selected foods (wheat, maize, beef, cheese, pasta, potatoes, carrots, rice, bread, chicken eggs, peanuts, beans, cabbage, apple, olive oil, and salmon).

Exclusion Criteria: Records with artificially contaminated food samples; Food samples stated for animal feed; Records with animals as experimental models.

Data Extraction

Relevant data was extracted from the text of the paper, tables, and figures or supplementary material of the eligible records. Data from graphs was extracted using WebPlotDigitalizer (version 5) by Automeris. Table 1 overviews the parameters outlined in the DIETxPOSOME databse and associated set of instruction for data extraction used by reviewers.

A total of 10 reviewers participated in data extraction, each responsible for independently extracting data from 14-17 articles. Subsequently, each reviewer cross-reviewed the data extracted by another reviewer, using a shared document to raise questions, offer suggestions, or clarify details. The second reviewer was able to see the first reviewer comments and reply/add information of interest. After this initial review, two reviewers performed a second revision across the dataset and uniformized the database.

Table S4: Parameters outlined in the DIETxPOSOME databse and associated set of instruction for data extraction.

**Cell Column**	**Description**	**Instructions**
**PMID**	PMID of research article	Each data row must be accompanied by PMID of the record of origin.
**DOI_path**	DOI of research article	Each data row must be accompanied by DOI path of the record of origin.
**Scientific_Name**	Scientific name of food	When available in the record.
**Food**<br>**Food_Variation**	Food sample analysed<br>Variation of food analysed	<u>Inclusion Criteria:</u><br>● Foods: Apple, Beans, Beef, Bread, Cabbage, Carrots, Cheese, Chicken Eggs, Maize, Olive Oil, Pasta, Peanuts, Potatoes, Rice, Salmon, Wheat<br>● Variation:<br><u>Bean</u>: Long bean, azuki beans, brown beans, black beans, white beans, lupine beans, green bean, fava bean, string bean, mung bean, kidney bean, pigeon pea, lima bean were also considered as beans.<br><u>Beef and salmon</u>: only meat (muscle) was considered.<br><u>Cabbage</u>: Chinese cabbage and napa cabbage were also considered as cabbage.<br><u>Maize</u>: Corn was inserted as maize.<br><u>Variation</u>: only meat (muscle) was considered.<br><br><u>Exclusion Criteria:</u><br>• Canned food samples were not considered.<br>• Juices or concentrates were not considered.<br>• In the case of beef/salmon, if the sample is cooked, marinades were not considered.<br>*Note: Following data extraction, food variation field was divided into food variation, food part and sample details.*
**Chemical**	Chemical compound	From the following list: 4,8-diMeIQx, 7,8-DiMeIQx, 8-MeIQx, Acenaphthene, Acenaphthylene, Acetamiprid, Aflatoxin B1, Altenuene, Alternaria toxins, Alternariol, Alternariol monomethylether, Altertoxin I, Anthracene, Arsenic, AαC, Beauvericin, Benz[a]anthracene, Benzo[a]pyrene, Benzo[b]fluoranthene, Benzo[g,h,i]perylene, Benzo[k]fluoranthene, Cadmium, Chlorpyrifos, Chlorpyrifos-methyl, Chrysene, Citrinin, Culmorin, Cypermethrin, Deltamethrin, Deoxynivalenol, Dibenz[a,h]Anthracene, Egocristinine, Egometrinine, Enniatin A, Enniatin A1, Enniatin B, Enniatin B1, Ergocornine, Ergocristine, Ergocroninine, Ergokryptine, Ergokryptinine, Ergometrine, Ergosine, Ergosinine, Ergotamine, Ergotaminine, Fluoranthene, Fluorene, Fumonisin B1, Fusaric Acid, Harman, Indeno[1,2,3-cd]pyrene, IQ, IQx, Lead, MeAαC, MeIQ, MeIQx, Mercury, Methomyl, Moniliformin, Naphthalene, Nivalenol, Norharman, Ochratoxin A, Patulin, Phenanthrene, PhIP, Propiconazole, Pyraclostrobin, Pyrene,

		Sterigmatocystin, T-2 toxin, Tebuconazole, Tentoxin, Tenuazonic acid, Zearalenone, λ-cyhalothrin.
**Weight_Basis**	Weight_basis of food sample	Fresh, dry and wet weight. Information was extracted according to what was stated in the record or by interpretation of methods sections. If information was unavailable, input was fresh weight. *Note: in the case of cereal grains/flours, even though they are known "dry foods", food samples were considered fresh unless stated otherwise in the record.*
**Units**	Units of chemical compound amount per quantity of food	
**Amount_Mean**<br>**Amount_Median**<br>**Amount_SD**<br>**Amount_SE**<br>**Amount_Min**<br>**Amount_Max**	Mean amount of the chemical compound (if applicable)<br>Median amount of the chemical compound (if applicable)<br>Standard deviation of amount of the chemical compound (if applicable)<br>Standard error of amount of the chemical compound (if applicable)<br>Minimum amount of the chemical compound (if applicable)<br>Maximum amount of the chemical compound (if applicable)	Food samples can be positive or negative samples for a given contaminant.<br>Depending on the data presentation on the record, different strategies were followed.<br><br>Case A: If the record clearly states the number of positive samples and a measured amount relating to positive samples (mean, median, range from minimum to maximum value).<br>● A first cell row should relate to positive sample values.<br>● For negative samples:<br>If information is available, that n samples are within LOD and LOQ:<br>Minimum: Should be inserted as LOD.<br>Maximum: Should be inserted as LOQ.<br>Mean: Should be inserted as <LOQ<br>If stated that n samples are below LOD:<br>Minimum: Should be inserted as 0.<br>Maximum: Should be inserted as LOD.<br>Mean: Should be inserted as <LOD.<br>There are cases, that the record considers samples <LOQ as negatives (it does not distinguish between LOD and LOQ).<br>Minimum: Should be inserted as 0.<br>Maximum: Should be inserted as LOQ.<br>Mean: Should be inserted as <LOQ.<br>Case B: If the record presents the results including both negative and positive samples.<br>● A single cell row should include total n samples.<br><br>However, for minimum amount:<br>The minimum amount should not be the lowest value of a positive sample, but the value used to consider negative samples. This value varies according to record and is sometimes present in subtitles (i.e. 0, LOD/2, or LOQ, etc). If not stated value should be considered as LOD.

**Num_Samples**	Number of Samples	
**Composite_Sample**		If Num_Samples corresponds to composite sample, cell value is Yes.
**Num_Primary_Samples**		Number of primary samples that form composite samples (if available, i.e. if Num_Samples=2 and corresponds to a composite of 5 samples, Num_Primary_Samples=10).
**LOD**	Limit of Detection	
**LOQ**	Limit of Quantification	
**Processing_State**	Food sample state	Raw, Cooked, Dried, Frozen
**Cooking_Type**	Type of cooking process	(if applicable, i.e. grilled, barbecued, etc.)
**Cooking_specs**	Specifications of the cooking processes	(if applicable)
**Country**	Country of sample origin or purchase	Author's country of origin was not considered.
**Method**	Method for quantification	
