# Supplementary material for: Exposure Scenarios for Estimating Contaminant Levels in Healthy Sustainable Dietary Models: Omnivorous vs. Vegetarian
Source: Foods. 2024 Nov 17;13(22):3659. doi: 10.3390/foods13223659 (PMC11593607; doi:10.3390/foods13223659)
Supplement: Supplementary file 1 [file foods-13-03659-s001.zip › Table S1.pdf]

Table S1: Nutritional composition of omnivorous and vegetarian dietary models, estimated based on TCAP data [25].

	Omnivorous dietary model	Vegetarian dietary model
**Energy** (kcal)	2338	2304
**Protein** (g)	104	95
**Fat** (g)	108	108
**Carbohydrates** (g)	222	215
**Fibre** (g)	31	44
**Vitamin A** (μg RE)	1834	1983
**Vitamin D** (μg)	13,5	2,11
**α-tocopherol** (mg)	15,5	11,3
**Thiamin** (mg)	1,2	1,3
**Riboflavin** (mg)	0,7	1,2
**Niacin** (mg NE)	30,7	40,5
**Vitamin B6** (mg)	1,5	1,9
**Vitamin B12** (μg)	2,6	1,86
**Vitamin C** (mg)	108	107
**Folate** (ug)	300	440
**Sodium** (mg)	2912	3081
**Potassium** (mg)	2408	3120
**Calcium** (mg)	849	1039
**Phosphorus** (mg)	1257	1606
**Magnesium** (mg)	278	418
**Iron** (mg)	8,7	15,4
**Zinc** (mg)	8,5	12,5
