# Supplementary material for: Exposure Scenarios for Estimating Contaminant Levels in Healthy Sustainable Dietary Models: Omnivorous vs. Vegetarian
Source: Foods. 2024 Nov 17;13(22):3659. doi: 10.3390/foods13223659 (PMC11593607; doi:10.3390/foods13223659)
Supplement: Supplementary file 1 [file foods-13-03659-s001.zip › Table S2.pdf]

Table S2: Detailed weighted mean values of PAHs for beef and salmon across different cooking types.

PAH (µg/kg)	Beef				Salmon			
	Barbecued	Fried	Grilled	Oven-Broiled	Raw	Grilled	Fried	Barbecued
**Ace**	3.06±0.43	0.01±0	0.33±0.12	0.01±0	1.39 ± 0	0.28 ± 0.1	0.35 ± 0.12	1.76 ± 0
**Acy**	37.66±5.08	0.01±0	0.16±0.06	0.01±0	0.27 ± 0.09	0.17 ± 0.06	4.94 ± 0	0.29 ± 0
**Ant**	15.73±1.93	0.3 ± 0	0.3 ± 0	4.4 ± 0	0.11 ± 0	0.3 ± 0	0.3 ± 0	4.4 ± 0
**B[a]A**	5.46±0.68	0.02±0	0.28±0.08	0.01±0	0.2 ± 0.02	0.01 ± 0.01	0.01 ± 0	1.16 ± 0.05
**B[a]P**	6.91±0.94	0.07±0.04	0.91±0.19	0.01±0	0.06 ± 0.01	0.01 ± 0	0.01 ± 0	0.76 ± 0.03
**B[b]F**	4.27±0.53	0.02±0	0.16±0.05	0.01±0	0.07 ± 0.01	0.01 ± 0	0.01 ± 0	0.91 ± 0.04
**B[ghi]P**	6.05±0.82	0.02±0	0.11±0.03	0.01±0	0.09 ± 0	0.01 ± 0	0.01 ± 0	0.55 ± 0.03
**B[k]F**	2.14±0.29	0.01±0	0.05±0.01	0.01±0	0.06 ± 0.01	0.01 ± 0	0.01 ± 0	0.24 ± 0.03
**Chr**	8.13±0.9	0.03±0.01	0.42±0.13	0.01±0	0.19 ± 0.02	0.04 ± 0.02	0.09 ± 0	2.3 ± 0.15
**D[a,h]A**	0.47±0.07	0.01±0	0.08±0.02	0.01±0	0.07 ± 0.01	0.01 ± 0	0.01 ± 0	0.05 ± 0.02
**Fla**	29.52±3.48	0.07±0.02	0.52±0.15	0.01±0	0.11 ± 0	0.29 ± 0.04	0.38 ± 0	8.3 ± 0
**F**	18.79±2.37	0.03±0.02	0.47±0.15	0.01±0	0.72 ± 0	1.43 ± 0	1.47 ± 0	5.46 ± 0
**IP**	4.71±0.65	0.01±0	0.13±0.04	0.01±0	0.09 ± 0	0.01 ± 0	0.01 ± 0	0.43 ± 0.07
**Phe**	60.53±6.1	0.19±0.05	0.4±0.16	0.01±0	1.81 ± 0	-	-	-
**P**	31.62±3.83	0.08±0.02	0.63±0.19	0.01±0	0.49 ± 0	2.1 ± 0	2.02 ± 0	27.01 ± 0
