# Supplementary material for: Exposure Scenarios for Estimating Contaminant Levels in Healthy Sustainable Dietary Models: Omnivorous vs. Vegetarian
Source: Foods. 2024 Nov 17;13(22):3659. doi: 10.3390/foods13223659 (PMC11593607; doi:10.3390/foods13223659)
Supplement: Supplementary file 1 [file foods-13-03659-s001.zip › Table S3.pdf]

Table S3: Distribution of individual HAAs in omnivorous (OMN) and vegetarian (VEG) models at the 25th, 50th, and 75th percentiles scenarios.

HAAs (µg per diet)	25th percentile		50th percentile		75th percentile	
	OMN	VEG	OMN	VEG	OMN	VEG
4,8-dMeIQx	0.02	0	0.02	0	0.02	0
7,8-dMeIQx	0.15	0	0.15	0	0.15	0
AαC	18.65	0	18.65	0	18.65	0
Harman	0.22	0	0.26	0	1.91	0
IQ	0.41	0	0.41	0	0.41	0
IQx	0.28	0	0.28	0	0.28	0
MeIQ	0.01	0	0.01	0	0.01	0
MeIQx	0.07	0	0.10	0	0.12	0
Norharman	0.16	0.01	0.17	0.01	0.55	0.01
PhIP	0.09	0	0.15	0	1.44	0
